# Histone Deacetylase Inhibitors in Clinical Studies as Templates for New Anticancer Agents

**DOI:** 10.3390/molecules20033898

**Published:** 2015-03-02

**Authors:** Madhusoodanan Mottamal, Shilong Zheng, Tien L. Huang, Guangdi Wang

**Affiliations:** 1 RCMI Cancer Research Center, Xavier University of Louisiana, New Orleans, LA 70125, USA; E-Mails: szheng@xula.edu (S.Z.); thuang@xula.edu (T.L.H.); 2 Department of Chemistry, Xavier University of Louisiana, New Orleans, LA 70125, USA; 3 College of Pharmacy, Xavier University of Louisiana, New Orleans, LA 70125, USA

**Keywords:** HDAC inhibitors, cancer, molecular modeling, clinical trials

## Abstract

Histone dacetylases (HDACs) are a group of enzymes that remove acetyl groups from histones and regulate expression of tumor suppressor genes. They are implicated in many human diseases, especially cancer, making them a promising therapeutic target for treatment of the latter by developing a wide variety of inhibitors. HDAC inhibitors interfere with HDAC activity and regulate biological events, such as cell cycle, differentiation and apoptosis in cancer cells. As a result, HDAC inhibitor-based therapies have gained much attention for cancer treatment. To date, the FDA has approved three HDAC inhibitors for cutaneous/peripheral T-cell lymphoma and many more HDAC inhibitors are in different stages of clinical development for the treatment of hematological malignancies as well as solid tumors. In the intensifying efforts to discover new, hopefully more therapeutically efficacious HDAC inhibitors, molecular modeling-based rational drug design has played an important role in identifying potential inhibitors that vary in molecular structures and properties. In this review, we summarize four major structural classes of HDAC inhibitors that are in clinical trials and different computer modeling tools available for their structural modifications as a guide to discover additional HDAC inhibitors with greater therapeutic utility.

## 1. Background

Cancer is a disease driven by genetic and genomic alterations such as amplifications, translocations, deletions, and point mutations. However, cancer development is also tied to epigenetic changes due to modifications such as DNA methylation and post-translational histone acetylations that can alter DNA accessibilities and chromatin structures without alterations in the DNA sequence. The basic unit of chromatin is the nucleosome, which comprises 147 base pairs of DNA superhelix wrapped around a histone core consisting of two copies each of the core histones [1]. Histones are the primary protein components of chromatin of five classes (H1, H2A, H2B, H3 and H4). H1 is a linker histone and the remaining are the core histones. The core plays an important role in establishing interactions between the nucleosomes and within the nucleosome particle itself [2,3]. The N-terminal tails of core histones are flexible and unstructured, but the rest are predominantly globular and well structured. Depending on the epigenetic modifications that occur in DNA and in histone tails, chromatin can adopt different conformational changes that control the activation or repression of gene transcription.

There are at least eight distinct types of histone post-translational modifications, namely acetylation, methylation, phosphorylation, ubiquitylation, sumoylation, ADP ribosylation, deamination and proline isomerization. It can be viewed as a regulatory code that resides in the pattern of post-translational modifications for which the histone amino terminal tails are the target. The N-ε-lysine acetylation and deacetylation of histone are controlled by two groups of enzymes: histone acetyltransferase (HAT) and histone deacetylase (HDAC). The balance between acetylation and deacetylation of histones or the reverse activities of HATs and HDACs regulate gene expression through chromatin modifications [4,5]. Histone acetylation by HAT plays a key role in transcriptional activation, whereas deacetylation of histones promotes transcriptional repression and silencing of genes. An excessive level of histone acetylation induces apoptotic cell death, whereas excessive level of histone deacetylation has been linked to cancer pathologies by promoting the repression of tumor regulatory genes. Disruption of HAT and HDAC activities has been associated with the development of a wide variety of human cancers [5]. HDAC inhibitors cause an increase of the acetylated level of histones, which in turn promote the re-expression of the silenced regulatory genes in cancer cells and reverse the malignant phenotype. Due to this effect, HDAC inhibitors have recently emerged as potential cancer therapeutic agents.

## 2. Classification of HDAC Family

In the human genome, eighteen HDAC family members have been identified and are grouped into four classes based on their homology to yeast HDACs. Classes I, II and IV are Zn^2+^-dependent metalloproteins, whereas Class III is a nicotinamide adenine dinucleotide (NAD^+^)-dependent enzyme. Class I family of HDACs consists of HDAC1, 2, 3 and 8 proteins sharing sequence homology with yeast reduced potassium dependency-3 (Rpd3), and are mainly located in the nucleus of the cells [6,7]. Class II family HDACs are homologous to the yeast histone deacetylases 1 (Hda1) and are further divided into two subgroups, Class IIA (HDAC4, 5, 7 and 9) and Class IIB (HDAC6 and 10). Unlike Class I family HDACs, Class II family HDACS are primarily localized in the cytoplasm; however depending upon the phosphorylation status they can be shuttled between the cytoplasm and nucleus [8,9]. HDAC11 is the only member of Class IV family localized in the nucleus. It has a unique structure but shares some of the sequences of Class I and II enzymes. HDAC11 has been implicated in the regulation of interleukin-10 expression [10,11], OX40L surface expression [12] and expression of the DNA replication licensing factor Cdt1 [13]. Class III family comprise of seven members and they share sequence homology with yeast silent information regulator-2 (Sir2) protein. Hence Class III family HDACs are also known as sirtuins (SIRTs), and the seven members of this family are SIRT1 through SIRT7. SIRTs are located in three important cellular compartments: nucleus, cytoplasm and mitochondrion [14]. Phylogenetically SIRTs are further divided into four classes (SIRT1, SIRT2 and SIRT3 belong to Class I, a sole member of SIRT4 to Class II, SIRT5 to Class III, and SIRT6 and SIRT7 to Class IV) [14,15]. Sirtuins have emerged as potential therapeutic targets for the treatment of various diseases, such as cancer, cardiovascular, aging and neurodegenerative related diseases [16,17,18,19]. A recent review has summarized the possibility of sirtuins, especially SIRT1 and SIRT2, for cancer therapy agents [20]. Table 1 summarizes the classification, cellular localization, protein size, some biological implications and crystal structure availability of HDACs. This review focuses on recent development of inhibitors of metal-dependent “classical” HDACs (Classes I, II, and IV) that are in clinical trials as anti-cancer agents, and different computer modeling tools for the development of HDAC inhibitors.

## 3. Histone Deacetylases and Cancer

HDACs play a major role in the epigenetic regulation of gene expression through their effects on the compact chromatin structure. In recent years, HDACs have become promising therapeutic targets with the potential to reverse the aberrant epigenetic states associated with cancer. Alterations in acetylation levels and overexpression of various HDACs in many cancer cell lines and tumor tissues have been reported [21]. Characterization of post-translational modifications to histone H4 in a comprehensive panel of normal tissues, cancer cell lines and primary tumors suggests that global loss of monoacetylation at Lys16 of histone H4 is a common hallmark of human cancer cells, implicating a critical role of HDAC activity in establishing tumor phenotypes [22]. In cancer pathological conditions where the classical HDACs are overexpressed, inhibitors of HDACs were found to be effective in reversing the malignant phenotype of transformed cells and have subsequently emerged as promising cancer therapeutic agents. HDAC inhibitors have the potential to disrupt multiple signaling pathways to inhibit tumor growth and induce apoptosis. HDAC inhibitors can not only target histones but have the ability to influence a variety of processes such as cell cycle arrest, angiogenesis, immune modulation and apoptosis by targeting nonhistone proteins [21,23]. Several nonhistone proteins have been identified as HDAC substrates with diverse biological functions and they include, transcription factors (E2F, p53, c-Myc, NF-κB), hypoxia-inducible factor 1 alpha (HIF-1α), estrogen receptor (ER α), androgen receptor (AR), MyoD, Chaperons (HSP90), signaling mediators (Stat3, Smad7), DNA repair proteins (Ku70), α-tubulin, β-catenin, retinoblastoma protein (pRb) and many others [24,25]. 

**Table 1:** Histone deacetylase enzymes: classification, amino acid size, cellular localization, physiological functions and crystal structure availability.

Metal Dependent					
Class	Members	Size (aa)	Cellular Localization	Physiological Function	X-ray Crystal
I	HDAC1	483	Nucleus	Cell survival and proliferation	Yes
	HDAC2	488	Nucleus	Cell proliferation, Insulin resistance	Yes (core domain)
	HDAC3	428	Nucleus	Cell survival and proliferation	Yes
	HDAC8	377	Nucleus	Cell proliferation	Yes
IIA	HDAC4	1084	Nucleus/Cytoplasm	Regulation of skeletogenesis and gluconeogenesis	Yes (catalytic & glutamine rich domains)
	HDAC5	1122	Nucleus/Cytoplasm	Cardiovascular growth and function, gluconeogenesis, cardiac myocytes and endothelial cell function	No
	HDAC7	912	Nucleus/Cytoplasm	Thymocyte differentiation, endothelial function, glucogenesis	Yes (catalytic domain)
	HDAC9	1069	Nucleus/Cytoplasm	Homologous recombination, thymocyte differentiation, cardiovascular growth and function	No (structure is known for aa 138–158)
IIB	HDAC6	1215	Cytoplasm	Cell motility, control of cytoskeletal dynamics	Yes (zinc finger and ubiquitin binding domains)
	HDAC10	669	Cytoplasm	Homologous recombination, Autophagy mediated cell- survival	No
IV	HDA11	347	Nucleus	Immunomodulators-DNA replication	No
**NAD+ Dependent**					
III	SIRT 1	747	Nucleus, Cytoplasm	Aging, redox regulation, cell survival, autoimmune system regulation	Yes (catalytic domain)
	SIRT 2	389	Nucleus	Cell survival-cell migration and invasion	Yes
	SIRT 3	399	Mitochondria	Urea Cycle, Redox balance, ATP regulation, metabolism, apoptosis and cell signaling	Yes
	SIRT 4	314	Mitochondria	Energy metabolism, ATP regulation, metabolism, apoptosis and cell signaling	No
	SIRT 5	310	Mitochondria	Urea cycle, Energy metabolism, ATP regulation, metabolism, apoptosis and cell signaling	Yes
	SIRT 6	355	Nucleus	Metabolic regulation	Yes
	SIRT 7	400	Nucleus	Apoptosis	No

Thus the disruption of multiple pathways by HDAC inhibitors and their lack of enzyme specificity cause additional complication to rational drug design for a specific disease state. In clinical studies several classes of HDAC inhibitors demonstrated potent anticancer activities with remarkable tumor specificity, such as cutaneous T-cell lymphoma and peripheral T-cell lymphoma [26,27,28,29].

To date, three HDAC inhibitors have been approved for cancer therapy by the US Food and Drug Administration (FDA). The first drug, Vorinostat (SAHA, Zolina), developed by Merck & Co. Inc. was approved in October 2006 for use in patients with cutaneous T-Cell Lymphoma (CTCL), a rare type of non-Hodgkin’s lymphoma of the skin. Vorinostat is structurally related to trichostatin A (TSA), a hydroxamic acid-containing natural product that was found to possess HDAC inhibitor activity and originally used as an antifungal drug. The second drug, romidepsin (Istodax, FK228, FR901228, depsipeptide), developed by Gloucester Pharmaceuticals (acquired by Celgene in 2009) was approved at the end of 2009, also for the treatment of T-cell lymphoma. Romidepsin is a unique natural product isolated from the cultures of *Chromobacterium violaceum*, a Gram negative bacterium isolated from a Japanese soil sample [30]. In June 2011, romidepsin was also approved for peripheral T-cell lymphoma (PTCL) in patients who have received at least one prior therapy. The third drug, belinostat (Beleodaq, PXD-101), developed by Spectrum Pharmaceuticals was approved on July 3, 2014 for the treatment of patients with relapsed or refractory peripheral T-cell lymphoma (PTCL) [31].

Over the past several years, a number of small molecule HDAC inhibitors have been subjected to clinical trials for various types of cancers. Based on their distinct chemical structure, these inhibitors can be grouped into four different classes, comprising hydroxamic acids, benzamides, cyclic peptides and short-chain fatty acids [32]. Vorinostat and belinostat belong to the hydroxamic acid class, and romidepsin is a member of the cyclic peptide class. The most widely explored class of HDAC inhibitors that have entered pre-clinical or clinical studies as anti-cancer agents are the hydroxamic acid-based compounds. Besides Vorinostat and belinostat, some of the novel hydroxamic acid based HDACi that are in different stages of clinical studies are abexinostat (PCI-24781), pracinostat (SB939), resminostat (RAS2410, 4SC-201), givinostat (ITF2357), quisinostat (JNJ-26481585), panobinostat (LBH589) and CUDC-101 [33]. Interestingly, HDAC inhibitors share common structural features so that they can properly interact with different portions of the catalytic channel of the enzyme. HDAC inhibitors generally consist of three parts in chemical structure with distinct pharmacophore features: (1) a zinc chelating group; (2) a spacer group; which is generally hydrophobic and (3) an enzyme binding group that confers specificity and is generally aromatic in character [34]. A range of natural and synthetic HDAC inhibitors have been characterized for their antitumor activities. Although not fully understood, the clinical activities of these compounds are thought to be mediated in part by the induction of histone acetylation where the chromatin configuration adopts a permissive or more open form for potential reactivation of aberrantly suppressed genes, leading to inhibition of cell proliferation, cell differentiation and apoptosis [35]. In the following sections, we describe the clinical development of different classes of HDAC inhibitors.

## 4. FDA Approved Drugs

To date, only three HDAC inhibitors have been approved by the FDA for the treatments of CTCL (vorinostat (SAHA) and romidepsin (Istodax)) and PTCL (belinostat (Beleodaq) and romidepsin). Currently all three drugs are being further evaluated for other diseases as well as in other hematological malignancies and solid tumors, either as a single agent or in combination with other drugs. The following subsections summarize the research done with these three drugs for various diseases.

### 4.1. Vorinostat

FDA approval of this hydroxamic acid based drug for the treatment of cutaneous manifestation of CCTL in patients with progressive, persistent or recurrent disease was based on Phase II clinical trials that enrolled 74 patients who had stage IB or higher CTCL. The objective response rate determined by direct evidence of clinical benefit was 30% [26]. For hematological malignancies, vorinostat can be given orally with a maximum tolerated dose of 400 mg once daily or 200 mg twice daily, but the dose level can be increased up to 600 mg in solid tumors [36]. Preclinical studies involving vorinostat have demonstrated its use as a potent radiosensitizer in human glioblastoma cell lines [37]. Vorinostat in combination with temozolomide and radiotherapy are currently in an ongoing clinical trial (NCT00731731) for treating patients with newly diagnosed glioblastoma multiforme (GBM). GBM is the most common and aggressive malignant brain tumor with very poor prognosis. Vorinostat showed potent apoptotic and anti-proliferative effect in both type I and type II human endometrial cancers by modifying the expression of specific genes related to the insulin-like growth factor-I (IGF-I) receptor signaling pathway [38]. In type I cell lines, vorinostat increased the IGF-IR phosphorylation, up-regulated PTEN and p21 expression, and reduced p53 and cyclin D1 levels. In type II cell lines, vorinostat up-regulated IGF-IR and p21 expression, and down-regulated the expression of total AKT, PTEN and cyclin D1. Interestingly, vorinostat hyperacetylated histone H3 in both type I and type II endometrial cancer cell lines, implying the role of histone H3 in endometrial cancer. Endometrial cancer is the most common gynecologic cancer that begins in the endometrium, the inner lining of the uterus, and these are classified into Type I and Type II groups, with type I being the most frequent [39,40]. In murine and human lung cancer cell lines and genetically engineered mouse lung cancer models, Vorinostat reduced cancer cell growth, cyclin D1 and cyclin E expressions, but increased p27 expression, histone acetylation and apoptosis [41]. Under hypoxia, radiosensitization by vorinostat in combination with capecitabine decreased colonogenicity *in vitro*, and inhibited tumor growth *in vivo* in xenograft models of colorectal carcinoma [42]. Currently vorinostat in combination with CHOP (cyclophosphamide, doxorubicin, vincristine, prednisone) that exhibits poor prognosis by itself is in clinical trials for treating patients with untreated PTCL [43]. Vorinostat has also been found to be a potent agent in the treatment of gastrointestinal (GI) cancer [44]. Vorinostat has also been implicated in having an effect on other types of cancers, such as brain metastasis, refractory colorectal, advanced solid tumors, melanoma, pancreatic, lung cancer and multiple myeloma. In terms of its target, vorinostat inhibits Class I, II and IV HDAC proteins, but not the NAD^+^-dependent Class III HDAC [45,46,47].

### 4.2. Romidepsin (Depsipeptide, ISTODAX)

The second HDAC inhibitor approved for the treatment of CTCL was based on two large phase II studies: a multi-institutional study based at the NCI in the US (71 patients), and an international study (96 patients) [27,28]. The treatment schedule was identical across both studies and the overall response rate was 34% in both studies. Romidepsin also induced complete and durable responses in patients with relapsed or refractory PTCL across all major PTCL subtypes, regardless of the number or types of prior therapies, with an objective response rate of 25%, which led to the approval of single agent romidepsin for the treatment of relapsed or refractory PTCL in the US [48]. Similarly, a phase II trial enrolling 47 patients with PTCL of various subtypes including PTCL NOS, angioimmunoblastic, ALK-negative anaplastic large cell lymphoma, and enteropathy-associated T-cell lymphoma also showed an overall response rate of 38% [49]. Romidepsin was also implicated in inhibiting the growth of non-small cell lung cancer (NSCLC) cells. A recent study concluded that romidepsin and bortezomib cooperatively inhibit A549 NSCLC cell proliferation by altering the histone acetylation status, expression of cell cycle regulators and matrix metalloproteinases [50]. Investigation of romidepsin for the treatment of inflammatory breast cancer (IBC), the most metastatic variant of locally advanced breast cancer, revealed that it potentially induced destruction of IBC tumor emboli and lymphatic vascular architecture [51]. Romidepsin, either as a single agent, or in combination with paclitaxel, effectively eliminated both primary tumors and metastatic lesions at multiple sites formed by the SUM149 IBC cell line in the Mary-X preclinical model [51]. A combination of depsipeptide and gemcitabine was tested in patients with advanced solid tumors including pancreatic, breast, NSCLC and ovarian and the study identified a dose level of 12 mg/m^2^ romidepsin and 88 mg/m^2^ gemcitabine for phase II trial [52]. In another phase I trial, romidepsin was evaluated in patients with advanced cancers including patients with thyroid cancer and identified tolerable doses for the treatment [53]. According to *clinicaltrials.gov*, romidepsin is currently being evaluated in nearly 30 studies, either as a single agent or in combination with other drugs for treating mainly T-cell lymphoma.

### 4.3. Belinostat (Beleodaq)

Approval of the third pan-HDAC inhibitor, belinostat was based on a multi-center, single arm BELIEF trial of 120 evaluable patients with PTCL that was refractory or had relapsed after prior treatment [54]. Among patients with histologically confirmed PTCL (*n =* 120), the overall response rate was 25.8%. Similar to other two FDA approved drugs, belinostat was also tested in Phase I and Phase II clinical trials for both solid and hematological cancers. For example, the response rate of belinostat was tested for a second line therapy in 13 patients with recurrent or refractory malignant pleural mesothelioma and identified two patients with stable disease [55]. A Phase II trial of belinostat in women with platinum resistant epithelial cancer (OEC) and micropapillary (LMP) ovarian tumors showed good drug tolerance in both patient groups [56]. Belinostat was also tested in patients with recurrent or refractory advanced thymic epithelial tumors and the response rate was 8% among the thymoma patients but found no response among thymic carcinoma patients [57]. A phase II multicenter study was undertaken to estimate the efficacy of belinostat for the treatment of myelodysplastic syndrome (MDS), a cancer in which the bone marrow does not make enough healthy blood cells [58]. However, this study met the stopping rule in the first stage of enrollment itself, hence the trial was closed to further accrual.

A Phase II study involving 29 women with recurrent or persistent platinum-resistant ovarian cancer was also conducted to evaluate the impact of belinostat in combination with carboplatin [59]. The overall response rate was 7.4% and the addition of belinostat to carboplatin had little activity in a platinum-resistant ovarian cancer patients. Phase II clinical activity of belinostat was also tested in combination with carboplatin and paclitaxel by enrolling 35 women with previously treated ovarian cancer [60]. Combination of these three drugs were reasonably well tolerated with an overall response rate of 43% and demonstrated clinical benefits in patients with OEC. In patients with relapsed or refractory acute myeloid leukemia (AML), the effect of belinostat was studied in a phase II clinical study and it was found that the effect of belinostast as a single agent is minimal in AML patients [61]. A phase 1/II trial of belinostat in combination with cisplatin (P), doxorubicin (A), and cyclophosphamide (C) in thymic epithelial tumors (TETs) showed that belinostat in combination with PAC was active and feasible in TETs [62]. A preclinical study of belinostat in three hepatocellular carcinoma cell lines (PLC/PRF/5, Hep3B and HepG2) showed that it can inhibit cell growth in a dose dependent manner and induce histone acetylation in all three cell lines [63]. Antileukemia activity of this compound as a single drug and in combination with all-*trans*-retinoic acid was characterized in promyelocytic leukemia HL-60 and NB4 cell lines, where belinostat can exert dose-dependent growth inhibitory or proapoptotic effects promoting cell cycle arrest at the G0/G1 or the S transition phase [64].

While three hydroxamic acid derivatives as HDAC inhibitors have been clinically approved, the indication is mainly CTCL, not any solid tumor form. So far, few ongoing clinical trials are designed to combat solid tumors, and the ones that have been completed had very limited therapeutic outcome with regard to using the HDAC inhibitors for treatment of nonhematological cancers. This persistent gap limits the utility of HDAC inhibitors, but more importantly, it calls for the discovery of more selective inhibitors that are also pharmaceutically more robust.

The exact reasons why HDAC inhibitors are more effective in hematological malignancies than in solid tumors are not well understood, but some observations suggest that the inhibitors having gone through clinical trials so far may not be sufficiently stable to reach solid tumor sites, and that they may not be target specific for solid tumors.

## 5. Different Classes of HDAC Inhibitors

### 5.1. Hydroxamic Acid Derivatives

Some of the initial clinical studies established that hydroxamic acid-based compound vorinostat is well tolerated in patients with CTCL, and observed promising anti-cancer activities in different types of cancer, such as diffuse large B-cell lymphoma, Hodgkin lymphoma, and other haematological and solid tumors [36,65,66,67]. Vorinostat was also found to inhibit tumor growth in rodent models of a variety of cancers (prostate cancer, leukemia, breast cancer, glioma, and lung cancer) [68]. Given the diverse anti-cancer activities of vorinostat, much effort has been made to explore hydroxamic acid derivatives as potential treatment for various cancers. Indeed, over the past several years, many hydroxamic acid derivative based HDACis have entered pre-clinical or clinical studies as anti-cancer agents with promising results, including abexinostat, pracinostat, resminostat, givinostat, panobinostat, and CUDC-101. These HDAC inhibitors are described below in more detail.

#### 5.1.1. Abexinostat (PCI-24781)

Abexinostat is a novel hydroxamate-based HDACi that showed broad spectrum anticancer activities in preclinical studies. As a single agent and in combination with the proteasome inhibitor bortezomib, abexinostat was tested in neuroblastoma cell lines [69]. Western blotting analysis showed the cleavage of caspase-3 and PARP, indicating apoptosis as a primary mechanism of action. Further studies with xenograft mouse models indicated increased survival among animals treated with a combination of abexinostat and bortezomib. This oral pan-HDACi was evaluated in patients with advanced solid tumors in two single agent phase I studies (PCYC-402 and CL1-78454-002), resulting in an optimal schedule for allowing higher doses in the next stage of the trials in solid tumors [70]. The effect of abexinostat, alone or in combination with conventional chemotherapy agents, was also tested *in vivo* in human soft tissue sarcoma (STS) models [71]. As a single-agent abexinostat showed modest effects on STS growth and metastasis, but marked inhibition effect was observed in combination with chemotherapy. In a phase I study, pazopanib (PAZ: a tyrosine kinase inhibitor approved for use in renal cell carcinoma) in combination with abexinostat was tested in patients with metastatic solid tumors, and the results presented at the ACSO annual meeting 2014 showed partial tumor response and disease stabilization. Further studies are being done and this trial is currently recruiting patients (clinical trial information, NCT01543763). Similarly a phase I study was done with abexinostat in combination with cisplatin in patients with advanced keratinizing nasopharyngeal carcinoma (NPC), leading to the identification of optimal doses for combination therapy (clinical trial information: ISRCTN96922360).

#### 5.1.2. Pracinostat (SB939)

Pracinostat is another hydroxamate-based HDAC inhibitor for which clinical trials have been carried out. A phase II study tested the activity and tolerability of pracinostat in patients with intermediate or high risk myelofibrosis (MF) where pracinostat was shown to have clinical benefit and modest activity in patients with MF [72]. In another phase II study, pracinostat was tested in advanced solid tumor patients [73]. The drug was well tolerated, but there was no clear relationship between the acetylated histone H3 changes and dose level or antitumor response. Pracinostat was also found to be well tolerated in children with refractory solid tumors [74].

#### 5.1.3. Resminostat

Resminostat was evaluated in a pharmacokinetics and pharmacodynamics phase I study for patients with advanced solid tumors, yielding a recommended phase II dose of 600 mg/day [75]. Low micro- molar concentrations of resminostat abrogated cell growth and strongly induced apoptosis in multiple myeloma (MM) cell lines [76]. Synergistic effects were observed when it was used in combination with melphalan, bortezomib and S-2209 [76]. In a Phase II SAPHIRE trial, resminostat was also tested in relapsed or refractory Hodgkin Lymphoma (HL) [77,78]. Assessment of disease status was carried out by computed tomography in combination with positron emission tomography (PET/CT). This study achieved clear objective responses in relapsed/refractory HL patients and showed excellent safety profiles in heavily pre-treated patient population. Resminostat was also tried in patients with advanced hepatocellular carcinoma (HCC), either alone or in combination with sorafenib [79]. The combination treatment provided a substantial overall survival (OS) benefit (median OS of 8.1 months) for advanced HCC patients who had developed progressive tumor disease under first-line sorafenib therapy. Resminostat is also in clinical trials for treating advanced colorectal carcinoma (NCT01277406).

#### 5.1.4. Givinostat

Givinostat is a hydroxamic acid-containing HDAC inhibitor which has shown clinical benefits in patients with Hodgkin’s lymphoma, chronic lymphocytic leukemia and multiple myeloma. A phase II study was conducted to evaluate the safety and efficacy of givinostat in patients with JAK2^V617F^ positive myeloproliferative neoplasms (MPN), a type of blood cancer [80]. Complete and partial responses were documented suggesting givinostat as a promising drug for further clinical investigation in patients with MPN. An *in vitro* study determined if givinostat and hydroxyurea induce synergistic cytotoxicity in JAK2^V617F^ cells [81]. At low doses, both givinostat and hydroxyurea potentiated the pro-apoptotic effects of each other in the JAK2^V617F^ HEL and UKE1 cell lines. As a single agent, givinostat and hydroxyurea induced 6.8%–20.8%, and 20.4%–42.4% cell death, respectively, whereas in combination of these two drugs the cell death was 35.8%–75.3%. This study suggested that a combined treatment with givinostat and hydroxyurea is a potential strategy for the management of JAK2^V617F^ myeloproliferative neoplasms. A phase I safety and pharmacokinetic trial in healthy males was also done with givinostat and identified the safe therapeutic dosing of givinostat [82]. In another multicenter, open-label phase II trial, patients with polycythemia vera (PV), unresponsive to the maximum tolerated doses (TMD) of hydroxycarbamide (HC), were treated with givinostat in combination with TMD of HC [83]. Complete or partial response was observed in 55% and 50% of the patients who received 50 or 100 mg of givinostat, respectively. This study showed that the combined use of givinostat and HC was safe and well tolerated, and clinically effective in HC-responsive PV patients.

#### 5.1.5. Panobinostat (LB589)

This hydroxamate-based panobinostat showed activity in clinical trials with different solid and heamatological cancers. The antitumor activity of panobinostat in patients with previously treated small-cell lung cancer (SCLC) was tested in a multicenter, nonrandomized phase II trial [84]. Although panobinostat was well tolerated and induced tumor shrinkage and sustained stable disease in SCLC, this study was prematurely closed because of a lack of activity. A phase I study investigated the effect of panobinostat in patients with primary myelofibrosis (PMF), post-essential thrombocythemia myelofibrosis (post-ET MF) and post-polycythemia vera myelofibrosis (post-PV MF) [85]. Panobinostat was well tolerated in MF patients with clinical improvements indicated by 100% reduction in palpable splenomegaly, and stable disease or near complete remission was observed in some patients. A phase I trial of panobinostat in 14 patients with advanced solid tumors was conducted in three cohorts. Even though stable disease (for ≥4 months) was observed in six patients, complete or partial responses were not observed in this study [86]. 

In another phase I trial in patients with advanced solid tumors or cutaneous T-cell lymphoma, good tolerance to panobinostat was observed when administered orally thrice in a week [87]. A multicenter, international Phase II study examined the safety and activity of panobinostat in 129 patients with relapsed/refractory Hodgkin’s lymphoma after autologous stem-cell transplantation, and observed tumor reductions in 74% of the patients with a 1 year survival rate of 78% [88]. In another phase II trial, panobinostat as a single agent was tested in red blood cell transfusion-dependent low or intermediate-1 risk MDS patients, but did not demonstrate a meaningful clinical activity [89]. A phase II trial of panobinostat in patients with low or intermediate-1 risk MDS observed only limited activity [90].

#### 5.1.6 CUDC-101

A recent study has shown simultaneous inhibition of HDAC and receptor tyrosine kinases (epidermal growth factor receptor—EGFR—and human epidermal growth factor receptor 2—HER2) in cancer cells, and displayed antiproliferative and proapoptotic activities *in vitro* as well as in drug-resistant *in vivo* tumor models [91]. Hence it has the potential to improve the treatment of heterogeneous and drug resistant tumors that cannot be controlled with singe-target agents. This synergistic inhibition was also tested in patients with advanced solid tumor using CUDC-101, and the drug was found to induce histone H3 acetylation in some of the patients. This study recommended a dose of 275 mg/m^2^ CUDC-101 for further clinical testing [92]. Then a phase 1b (expansion) was conducted to further evaluate the safety and tolerability of CUDC-101 in patients with diverse cancers (advanced breast, gastric, head and neck, NSCLC or liver cancer), The drug was found to be well tolerated in these patients and exhibited antitumor activity [93,94]. Table 2 summarizes all the hydroxamic acid based HDAC inhibitors as potential therapeutics for various cancers that were in clinical trials.

### 5.2. Benzamide Derivatives

Benzamide containing HDAC inhibitors are another class of compounds that showed both *in vitro* and *in vivo* anticancer activities. Among them mocetinostat (MGCD0103) and entinostat (MS-275) are two examples of benzamide derivatives that had been taken to clinical trials.

**Table 2:** Hydroxamic acid based HDAC inhibitors in clinical trials.

Hydroxamic Acid Based HDAC Inhibitors (HDACi)	HDAC Specificity (Class)	*In Vitro* Potency	Combination	Cancer Types	Reference
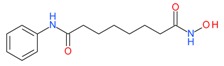 Vorinostat (SAHA)	I and II	nM	Temozolomide + radiation	Glioblastoma Multiforme (GBM)	[95]
			CHOP	Peripheral T-cell lymphoma (PTCL)	[43]
			-	Gastrointestinal(GI)	[44]
			Whole brain radiation	Brain metastasis	[37]
			5-fluorouracil/leucovorin(5FU/LV)	Refractory colorectal and solid tumors	[96,97]
			Hydroxychloroquine	Advanced solid tumors	[98]
			Marizomib	Melanoma, Pancreatic and Lung cancer	[99]
			Bortezomib	Multiple myeloma	[100]
			5-fluorouracil	Metastatic colorectal	[101]
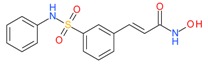 Belinostat (Beleodaq)	I and II	μM	-	Malignant pleural mesothelioma	[55]
			-	Epithelial & microcapillary ovarian cancers	[56]
			-	Thymic epithelial tumor(TETs)	[57]
			-	Myelodysplastic syndrom (MDS)	[58]
			Carboplatin	Platinum resistant ovarian cancer	[59]
			Carboplatin + Paclitaxel	Ovarian cancer	[60]
			-	Acute myeloid leukemia (AML)	[61]
			Cisplatin + doxorubicin + cyclophosphamide	Thymic epithelial tumors	[62]
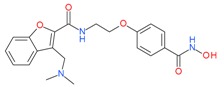 Abexinostat(PCI-24781)	I and II	nM	-	Advanced solid tumors	[70]
			Pazopanib	Metastatic solid tumor	[95]
			Cisplatin+radiation	Nasopharyngeal carcinoma (NPC)	[102]
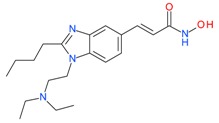 Pracinostat (SB939)	I, II and IV	μM	-	Myelofibrosis(MF)	[72]
			-	Advanced solid tumors	[73]
			-	Refractory solid tumors	[74]
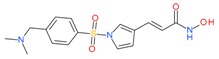 Resminostat	I and II	μM	-	Advanced solid tumors	[75]
			-	Relapsed/refractory Hogdkin Lymphoma (HL)	[77,78]
			or Sorafenib	Advanced hepatocellular carcinoma (HCC)	[79]
			-	Colorectal carcinoma	[95]
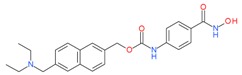 Givinostat (ITF-2357)	I and II	nM	-	Myeloproliferative neoplasms(MPN)	[80]
			Hydroxycarbamide	Polycythemia vera	[83]
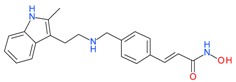 Panobinostat	I and II	μM	-	Small cell lung cancer (SCLC)	[84]
			-	Myelofibrosis(MF)	[85]
			-	Advanced solid tumors	[86]
			-	Cutaneous T-cell lymphoma	[87]
			-	Relapsed/refractory hogdkins lymphoma	[88]
			-	Myelodysplastic syndrome (MDS)	[89]
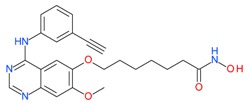 CUDC-101	I and II	nM	-	Advanced solid tumors	[92–94]

#### 5.2.1. Mocetinostat (MGCD0103)

This benzamide derivative HDAC inhibitor is selective for both Class I and IV histone deacetylases [103]. A phase 1 trial of mocetinostat in patients with leukemia or myelodysplastic syndromes (MDS) showed the drug was safe and exhibited antileukemia activity in these patients [104]. Three patients achieved a complete bone marrow response (blasts ≤ 5%) too. Mocetinostat was also tested in pancreatic cancer cell lines, and found dose-dependent growth arrest, cell death and cell cycle arrest. This effect was found to be enhanced when treated in combination with MC1568 (Class IIA selective HDACi) or tubastatin A (HDAC6 selective inhibitor) [105]. A phase II clinical trial in patients with chronic lymphocytic leukemia (CLL) also demonstrated efficacy with manageable side effects profile [106]. In patients with advanced solid tumors, mocetinostat inhibited HDAC activity and induced Histone H3 acetylation in peripheral white blood cells from these patients, and the trial identified a dose level of 45 mg/m^2^/day for Phase II studies [107]. The safety and efficacy of this compound was tested in patients with relapsed classical Hodgkin’s Lymphoma during a phase II clinical trial [108]. Even though the treatment showed promising clinical activity with manageable toxicity in patients with relapsed classical Hodgkin’s lymphoma, four patients died during the study, of which two might have been treatment-related deaths. As a result, this study has been terminated (clinical trial identifier: NCT00358982).

#### 5.2.2. Entinostat

Many clinical studies have investigated the activity of entinostat in many cancer cells, which include non-small cell lung cancer, breast cancer, lymphoblastic leukemia, renal cell cancer, colon cancer, metastatic melanoma and more. It is a Class I selective HDAC inhibitor and is well tolerated either as a single agent or in combination with other drugs [109]. For example, in a phase I trial, entinostat in combination with 13-*cis*-retinoic acid (CRA) was tested to determine the safety, tolerability, and the pharmacokinetic/pharmacodynamic profiles of entinostat and CRA in advanced solid tumors. While objective responses were not achieved, the combination drug was well tolerated and prolonged stable disease occurred in patients with prostate, pancreatic, and kidney cancer. In a randomized phase II trial to evaluate the effect of erlotinib with or without entinostat in advanced state NSCLC patients [110]. No improved outcome of patients in the overall study population was observed when compared with erlotinib monotherapy. Similarly, a placebo controlled randomized phase II study evaluated the effect of entinostat alone or combined with the aromatase inhibitor exemestane in breast cancer patients [111]. This study showed that a combination therapy of entinostat and exemestane is well tolerated and demonstrated clinical activity in patients with ER+ advanced breast cancer. Another phase I trial tested entinostat in patients with refractory solid tumors and lymphomas [112]. Prolonged disease stabilization was seen in some patients, and the drug was well tolerated and demonstrated antitumor activity.

### 5.3. Short Chain Fatty Acids

These compounds represent another class of HDAC inhibitor with simple structures that showed clinical potential in various studies. Valproic acid and phenylbutyrate are two well characterized compounds that belong to this class of compounds. They both display HDAC inhibition for Class I and IIa HDACs, but they tend to be less potent in inhibiting the HDAC activity than the hydroxamic acid based HDAC inhibitors.

#### Valproic Acid

This compound has entered in clinical trials as a single agent as well as in combination with other drugs [113]. In a phase I study, valproic acid (VPA) was tested in pediatric patients with refractory solid or central nervous system (CNS) tumors [113]. Increased histone acetylation in peripheral blood mononuclear cells was documented in 50% of patients studied, and the drug was well tolerated when administered three times daily to maintain a through concentration. In a pilot phase II study, VPA was also tested for the treatment of neuroendocrine tumors (NETs) and also to determine whether VPA can induce Notch 1 signaling *in vivo* [114]. Overall treatment with VPA was well tolerated in patients with NETs and was found to activate Notch1 signaling *in vivo*, suggesting its role in treating patients with low grade neuroendocrine carcinoma. 

VPA was also tested in combination with other drugs for the treatment of various cancers [115,116,117,118]. For example, a phase I study of the combination of bevacizumab (anti-angiogenic agent) and VPA was conducted in patients with advanced cancers, and demonstrated that the combination of bevacizumab and VPA is safe in patients with colorectal, prostate, and gastroesophageal cancers with ≥ 6 months of stable disease [115]. VPA was also tested in advanced stage NSCLC patients in combination with 5-aza-2'-deoxycytidine (decitabine). This combination therapy was found to be effective in reactivating hypermethylated genes as demonstrated by re-expressing fetal Hb, but was limited by unacceptable neurological toxicity at a relatively low dosage [116]. VPA in combination with S-1, an oral fluoropyrimidine derivative consisting of 5-fluorouracil, was tested in pancreatobiliary tract cancers and showed manageable safety and preliminary antitumor activity in these patients [117]. Table 3 summarizes the benzamide, short chain fatty acid, and cyclic peptide HDAC inhibitors and their respective activities against various cancers tested in clinical trials.

**Table 3:** Benzamide, short chain fatty acid and cyclic peptide based HDAC inhibitors in clinical trials.

HDACi	HDAC Specificity (Class)	*In Vitro* Potency	Combination	Cancer Types	Reference
Benzamide Based HDAC Inhibitors (HDACi)					
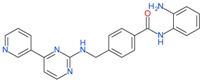 Mocetinostat (MGCD0103)	I and IV	μM	-	Leukemia	[104]
			-	Myelodysplastic syndrome (MDS)	[104]
			-	Chronic lymphocytic leukemia (CLL)	[106]
			-	Advanced solid tumors	[107]
			-	Relapsed Hodgkin’s lymphoma	[108]
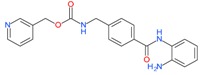 Entinostat (MS-275)	I	μM	13-cis retinoic acid(CRA)	Advanced solid tumors	[109]
			Erlotinib	NSCLC	[110]
			Exemestane	Breast cancer	[111]
			-	Refractory solid tumors and lymphoma	[112]
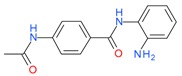 Tacedinaline (CI994)	I	μM	*-*	Advanced solid tumor	[119]
Short Chain Fatty Acid Based HDAC Inhibitors (HDACi)					
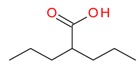 Valproic acid	I	mM	-	Refractory solid or central nervous system (CNS) tumors	[113]
			-	Neuroendocrine tumors(NET)	[114]
			Bevacizumab	Colorectal, Prostate, Breast, melanoma	[115]
			Decitabine	NSCLC	[116]
			S-1	Pancreatobiliary	[117]
			Hydralazine	Solid cancer	[118]
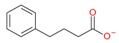 Phenylbutyrate	I and II	mM	-	Refractory solid tumor or lymphoma	[95]
			-	Recurrent brain tumor	[95]
			Azacitidine	Acute myeloid leukemia or MDS	[95]
			Azacitidine	Prostate cancer	[95]
			Azacitidine	NSCLC	[95]
Cyclic Peptide Based HDAC Inhibitors (HDACi)					
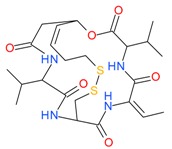 Romidepsin (Depsipeptide)	I	nM	-	Relapsed or refractory PTCL	[48,49]
			Bortezomib	NSCLC	[50]
			Abraxane	Inflammatory breast cancer	[95]
			Gemcitabine	Pancreatic, Breast, NSCLC, Ovarian	[52]
			-	Thyroid cancer	[53]

## 6. Natural HDAC Inhibitors

A large number of HDAC inhibitors are of natural origin. Hydroxamic acid-based trichostatin A (TSA) was one of first natural hydroxamate compounds isolated from the actinomycete *Streptomyces hygroscopicus* that was found to inhibit HDACs at IC_50_ less than 10 nM, with over 300-fold selectivity against Class IIa HDACs [120,121]. Another natural hydroxamate found to have anti-proliferative effects against various human tumor cells is amamistatin, isolated from *Nocardia asteroides* [122,123]. Short-chain fatty acids, such as sodium butyrate, the byproduct of anaerobic microbial fermentation inside the gastrointestinal tract, have been found to inhibit different classes of HDAC [124]. Both TSA and sodium butyrate downregulate the expression of Bcl-2 and induce apoptosis in lymphoma cells [125]. Short chain fatty acids like butyrate and propionate have been shown to increase apoptosis of neutrophils through HDAC inhibition [126]. Both propionate and butyrate based compounds are being tested in clinical trials for many diseases. Natural cyclopeptide FR235222 isolated from the fermentation broth of *Acremonium* sp. caused accumulation of acetylated histone H4, inhibition of human leukemia cell (U937) proliferation, and cell cycle arrest in the G1 phase via p21 [127]. Other natural cyclopeptides that have been demonstrated to act as HDAC inhibitors are chlamydocin from *Diheterospora chlamydosporia* [128], apicidin from *Fusarium* sp. [129] and azumamide A-E from the marine sponge *Mycale izuensis* [130,131], and the microbial metabolite trapoxin [132]. In addition to romidepsin, some other natural products that belong to the depsipeptide class with antitumor activities are largazole from cyanobacterium *Symploca* sp [133] spiruchostatin from *Pseudomonas* [134] and burkholdacs and thailandepsin from *Burkholderia thailandensis* [135]. They all exhibited prominent antitumor activity against various mammalian solid tumors. Several analogues of chlamydocin, largazole and apicidin also demonstrated anticancer activities in various cancers. Stilbene-based HDAC inhibitors such as resveratrol from red grapes have demonstrated promising activities for the prevention and treatment of cancer [136]. Resveratrol and its analogue piceatannol from blueberries are also known to be SIRT1 activators. Similarly several organosulfur compounds such as diallyl disulfide and allyl mercaptan from garlic [137,138] and sulforaphane from broccoli sprouts [139] inhibit HDAC activity in various cancer cells including colon, prostate and breast cancer cells. 

**Figure 1:**
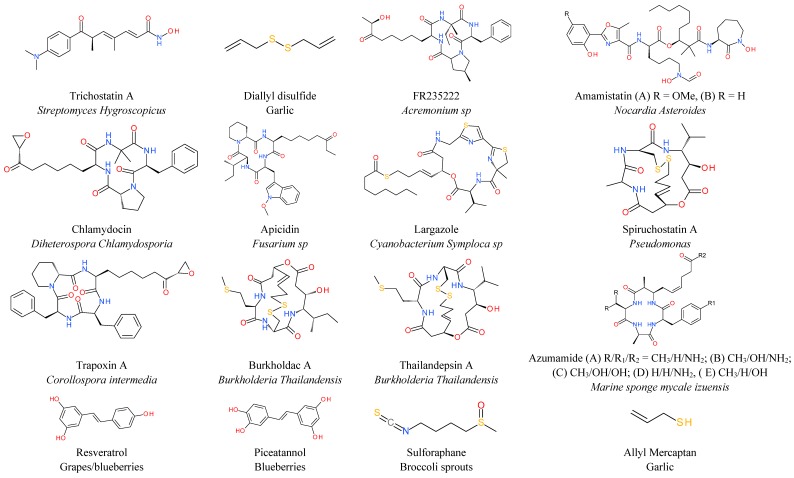
Natural product HDAC inhibitors and their sources.

Various other natural products from different sources are also found to inhibit HDAC activity. Two dimensional drawing of all the compounds discussed here are depicted in Figure 1.

## 7. Miscellaneous

### 7.1. Thioester Based HDACi

Thioesters are used for prodrug strategies. Largazole is a depsipeptide with a thioester moiety purified from marine cyanobacteria and it is a Class I selective HDAC inhibitor. Largazole upon protein-assisted hydrolysis liberates the bioactive largazole thiol. Disulfide prodrug strategy to modulate largazole-based compounds resulted in enzymatic activities comparable to the natural product largazole [140]. KD5170, a mercaptoketone-based Class I and II HDAC inhibitor which is another thioester prodrug demonstrated broad spectrum cytotoxicity against a range of human tumor-derived cell lines. In the proposed mechanism of action, the thioester prodrug undergoes hydrolysis to generate mercaptoketone that coordinates Zn^2+^ in a bidentate or monodentate fashion in the active site of HDACs [141,142]. Similarly, thioester derivatives of the natural product psammaplin A, a prodrug requiring reduction of its disulfide to the corresponding thiol monomer for the potential inhibition of HDACs, exhibited both potent cytotoxicity and enzymatic inhibitory activity against recombinant HDAC1 [143]. Among the three thioester compounds that contain an oxime or methyloxime or ketone moiety on the linker that connects the cap group, the ketone containing compound was found to be highly potent against recombinant HDAC1, displaying an IC_50_ of 5 nm. Preliminary investigation discounted the hydrolysis of thioester under the buffered conditions of the assay and direct cleavage of the acetyl group by the deacetylase enzyme. So in this case, rather than acting as a prodrug, the authors state that it is highly plausible that the thioacetate group can function as a potent zinc-binding group.

### 7.2. Epoxide Based HDACi

Epoxides are another known group of inhibitors of zinc dependent HDAC enzymes. Epoxide bearing natural compounds such as trapoxins and depudecin are reported to form covalent bonds with HDACs [144]. The HDAC activity of these compounds occur at micromolar to nanomolar concentrations [145,146]. Depudecin is a microbial metabolite containing two epoxide groups, whereas trapoxin has only one epoxide group. 1-Alaninechlamydocin isolated from *Tolypocladium sp*. showed potent antiproliferative/cytotoxic activities in human pancreatic cancer cell lines MIA PaCa-2 at low nanomolar concentrations and induced G2/M cell cycle arrest and apoptosis [147]. It exhibited comparable potency to the cyclic epoxytetrapeptide HDAC inhibitor trapoxin A, but greater potency than SAHA and apicidin in pancreatic carcinoma cell line MIA PaCa-2.

### 7.3. Electrophilic Ketone Based HDACi

**Figure 2:**
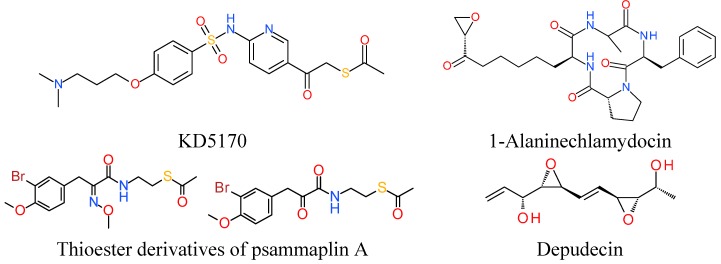
Structures of thioester- and epoxide-based HDAC inhibitors.

Trifluoromethyl ketones are known to be readily hydrated and have been reported as potent inhibitors of aspartyl, cysteine and serine proteases, as well as zinc dependent enzymes. Trifluoromethyl ketones attached to aromatic amides showed micromolar inhibitory activities as HDAC inhibitors in breast and fibrosarcoma cell lines [148]. Similarly cyclic tetrapeptides containing trifluoromethyl and pentafluoromethyl ketones as zinc binding functional groups were also found to be potent HDAC inhibitors with promising anticancer activities [149]. Fluorinated ketones are considerably more electrophilic because of the presence of strong electron withdrawing effect of the fluoride. Therefore trifluoromethyl ketones are readily hydrated in aqueous media at physiological pH. Trifluoromethyl ketones containing a thiophene linker have been reported as Class IIa selective HDAC inhibitors. A recent study demonstrated that the trifluoromethyl ketone moiety served as a potent zinc binding group [150]. The study also identified silanediol as a zinc binding group with potential for future development of non-hydroxamate Class I and Class IIb HDAC inhibitors. Figure 2 shows structures of some of the thioester and epoxide compounds that are discussed here.

## 8. Toxicity in Clinical Trials

As with any class of anticancer agents, HDAC inhibitors are also associated with toxicities. The most common grade 3 and 4 adverse events observed with the use of HDAC inhibitors were thrombocytopenia, neutropenia, anemia, fatigue and diarrhea [48,56,74,86,108,109]. In some cases, HDAC-induced thrombocytopenia can be rapidly reversible upon withdrawal of the drug [151]. Nausea, vomiting, anorexia, constipation and dehydration were also seen in patients receiving HDAC inhibitors. Deaths have been reported in clinical studies involving HDAC inhibitors. For example, when mocetinostat was tested in patients with relapsed Hodgkin’s lymphoma four patients died, of which two were treatment-related deaths [108]. Similarly deaths were also reported in clinical trials involving vorinostat [66], givinostat [152] and many other HDAC inhibitors. Thus more studies are needed to determine the toxicity of HDAC inhibitors before a clinical trial can be done, to minimize the cytotoxic effects in patients.

## 9. Basic Structure of Zinc Binding HDAC Inhibitors

**Figure 3:**
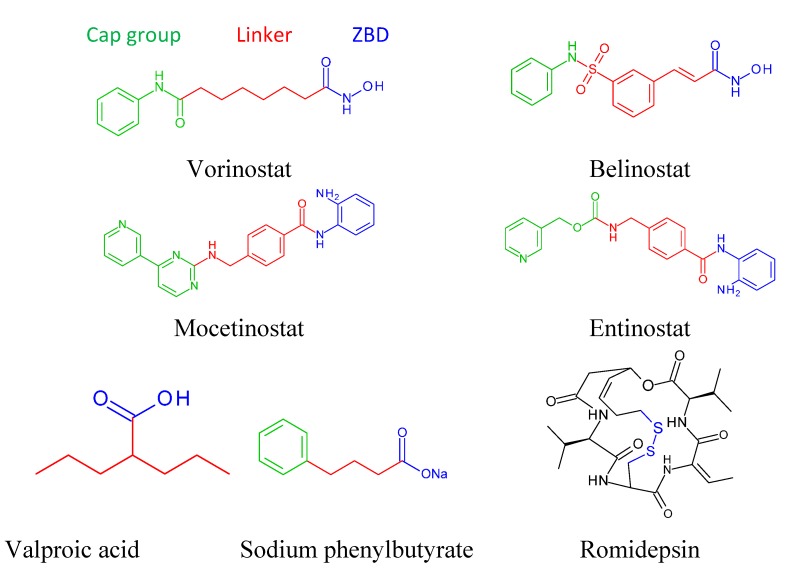
Structure of representative HDAC inhibitors and their pharmacophores. The cap group, linker and the zinc binding domain (ZBD) are colored green, red and blue, respectively.

As discussed here, a number of structurally distinct classes of HDAC inhibitors (hydroxamic acid, benzamide, cyclic peptide, short chain fatty acid) have been tested in clinical trials. Interestingly, most of the zinc-dependent HDAC inhibitors have common pharmacophores consisting of three distinct domains: (1) cap group or a surface recognition unit, usually a hydrophobic and aromatic group, which interacts with the rim of the binding pocket; (2) zinc binding domain (ZBD), such as the hydroxamic acid, carboxylic acid or benzamide groups, which coordinates to the active site of Zn^2+^ ion; and (3) a saturated or unsaturated linker domain with linear or cyclic structure, that connects the cap group to the ZBD [153]. Crystallographic analyses of HDAC in complex with hydroxamate compounds have revealed that the capping group is solvent exposed and interacts with the amino acids near the entrance of the active site, whereas the metal binding moiety resides in the interior of the protein and form complexes with the metal ion [34,154,155,156]. The linker serves to position the capping group and the metal binding domain appropriately for providing high affinity interactions with the proteins. Figure 3 shows the pharmacophoric summary and structure of a few selected HDAC inhibitors.

Variations in any or all three domains have variably contributed to the potency and selectivity in various HDAC inhibitors. In the case of the metal binding moiety, the functional groups contain hydroxamic acid, benzamides, thiols, ketones or epoxides. Comparing clinically important three drugs, vorinostat, entinostat and valproic acid that contain a hydroxamate, benzamide and a carboxylate metal binding moiety, respectively, a drastic change in the IC_50_ value was observed, when the hydroxamate (110–370 nM) [157,158] was changed to a benzamide (2 μM) [159] or a carboxylate (50 μM) [160]. Thus the presence of a carboxylate acid or a benzamide resulted in reduced inhibitory activity, perhaps due to their weaker metal-binding capacity than a hydroxamate group. Other studies also confirmed that hydroxamic acid is generally a more potent HDAC inhibitor than carboxylic acid [161]. Modification of the linker group, with different chain length, saturated or unsaturated hydrocarbons, including cyclic hydrocarbons have also displaced variations in the inhibitory activity. As a result, HDAC inhibitor design has involved these three modifications, as evident from several articles and reviews [33,162,163]. Thus finding the optimal structural requirements for HDAC inhibition is essential for developing more potent and specific inhibitors of different isoforms of HDAC.

## 10. Mechanism of action of HDAC inhibitors

HDAC inhibitors increase the level of histone acetylation and the mechanism for their antiproliferative effect is clearly associated with inhibition of HDAC activity. However, this effect alone is not sufficient to confer activity, because several trials have demonstrated increased histone acetylation in tumor samples despite little clinical effect [107,164]. HDACs can not only act on and modify histones, but also have many different cellular substrates and target proteins, including proteins that are involved in tumor progression, cell cycle control, apoptosis, angiogenesis and cell invasion. Thus HDAC inhibitors exert multiple cellular effects and the mechanism of action includes cell cycle arrest, activation of apoptotic pathway, induction of autophagy, reactive oxygen species generation, and angiogenesis.

HDACi mediated tumor cell death is mainly due to induction of apoptosis, which occurs through intrinsic (mitochondrial) or extrinsic (death receptor) pathways, both of which lead to caspase activation and cell death. Extrinsic pathway is initiated by binding of ligands, such as Fas ligand (FasL), tumor necrosis factor (TNF) and TNF-related apoptosis-inducing ligand (TRAIL) to their respective cell surface death receptors (DR), whereas intrinsic pathways are activated by disruption of mitochondrial membranes by cellular stresses such as chemotherapy, ionizing radiation, and withdrawal of growth factors [165]. Suberic bishydroxamate induces apoptosis in melanoma cells by the upregulation of Bim, Bax, Bak, while down regulating the expression of anti-apoptotic X-linked inhibitor of apoptosis, B-cell lymphoma-extra-large (Bcl-xL) and myeloid cell leukemia 1 (Mcl-1) [166]. Vorinostat treatment caused the general transcriptional induction of BH3-only pro-apoptotic protein encoding genes (Bad, Bim, Bix, Noxa), the multi-domain pro-apoptotic gene BAK1 and genes encoding death effector components downstream of mitochondrial damage (Diablo, Apaf1, Casp9, HtrA2 and CytC) in transformed fibroblasts [167]. Besides, the pro-survival genes, such as Bcl2A1, Bcl2L1 (encoding Bcl-xL) and Bcl2L2 (encoding Bcl-w), were concomitantly repressed in these cells. HDACi upregulated the expression of pro-apoptotic proteins Bmf, Bid, and Bim that belong to the Bcl2 family, and down regulated the expression of the anti-apoptotic proteins of the Bcl2 family such as Bcl2 and Bcl-x [21]. 

HDACi can also induce cell cycle arrest at G1/S or G2/M transition, leading to differentiation and/or apoptosis. HDACi-mediated increase in CDK inhibitor p21^WAF1/CIP1^ expression leads to cell cycle arrest at G1/S [168]. Silencing of HDAC3 has been found to induce the expression of p21^WAF1/CIP1^ and cell cycle arrest in the G2/M phase in colon cancer cells [169]. Vorinostat was found to promote cell cycle arrest at G1/S and G2/M and subsequent apoptosis of leukemic K562, HL60 and THP1 cells [170]. 

In another mechanism of action, HDACi can block tumor angiogenesis by inhibition of hypoxia inducible factors (HIF). Hypoxia upregulates gene expression of VEGF by stabilizing the transcription factor HIF 1α and tumor suppressor gene Von Hippel Lindau (VHL) degrades HIF 1α. Under hypoxic conditions trichostatin A (TSA) has been shown to upregulate VHL and p53 while downregulating VEGF and HIF 1α to block angiogenesis [171]. HDACi also contribute to the anti-angiogenic pathway by disrupting Hsp90 mediated chaperone function and exposing HIF 1α to proteosomal degradation [172].

HDAC inhibitors indirectly damage DNA by inducing changes in chromatin conformation upon histone acetylation that might expose the DNA to UV rays, ionizing radiation, ROS and chemotherapeutic genotoxic chemicals. This complex biochemical reaction can eventually lead to double strand breaks (DSBs) in DNA. The pan HDACi, vorinostat was shown to induce DSBs in normal (HFS) and cancer (LNCaP, A549) cells [173]. Normal cells in contrast to cancer cells repair the DSBs despite continued culture with vorinostat, whereas in transformed cells the level of biomarker of DBSs in DNA, phosphorylated histone variant γH2AX, increased with continued culture with vorinostat. DSBs are repaired by two independent pathways, homologous recombination (HR) and non-homologous end joining (NHEJ). HDACi can downregulate the levels of DNA repair proteins, such as Ku70 and Ku86 that are involved in NHEJ pathway [174,175]. Similarly HDACi suppressed the gene expression of DNA repair proteins like RAD51, BRCA1 and BRCA2 [176].

**Figure 4:**
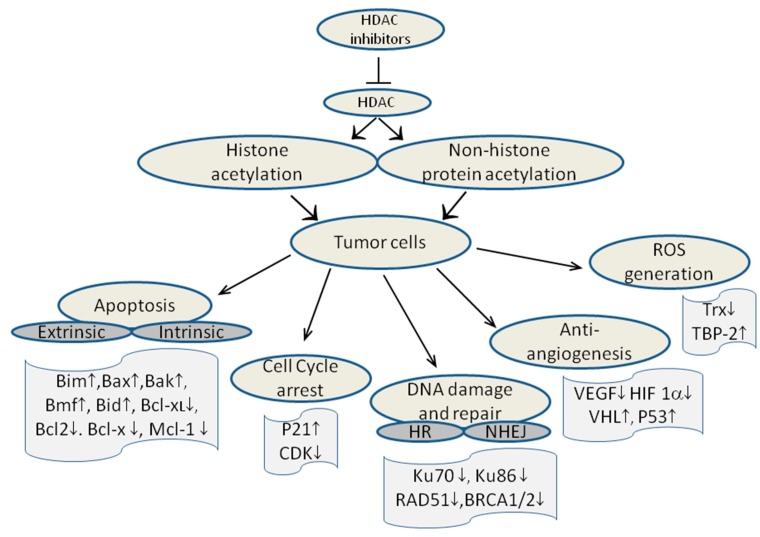
Multiple anti-tumor pathways activated by HDACi. Extrinsic and intrinsic refer to two apoptosis pathways, and HR and NHEJ refer to two DBS repair pathways.

Generation of reactive oxygen species (ROS) is another key event in HDACi induced cell death, causing DNA damage. Free radical scavengers like N-acetylcysteine reduce ROS generation which in turn abrogates HDACi mediated cell death [173,177]. HDACi increase ROS production through downregulation of thioredoxin (Trx), a thiol reductase that acts as a scavenger of ROS, and through upregulation of thioredoxin binding protein-2 (TBP-2), a protein that binds to Trx and blocks its reducing activity [178]. Treatment with vorinostat induced TBP-2 expression followed by suppression of Trx expression [179]. Together, these multifaceted mechanisms by which HDACi act upon cancer cell survival and death are depicted in Figure 4.

## 11. How to Obtain Novel HDAC Inhibitors?

In addition to the four well-known structural classes of HDAC inhibitors, there exist other HDAC inhibitors with different zinc binding groups, including thioesters, epoxides, epxoyketones, thiols, dithiols, ketones, hydroxypyridinethiones and hydroxypyridone. Many other structural classes that can inhibit HDAC activity may exist as well. How do we identify novel compounds that belong to these structural classes or to entirely new structural classes that can act as an anticancer agent by inhibiting the HDAC activity? Traditional high-throughput screening of libraries of compounds to identify potential inhibitors is an important and effective tool commonly used in pharmaceutical industry. In this method, tens to hundreds of thousands of small molecules are tested against a given assay to discover various novel drugs. However, this approach can be very expensive and resource intensive. In this regard, a variety of computational techniques can help to reduce the size of chemical library by focusing on those compounds that are predicted by *in silico* modeling to be most likely active. They include both structure-based and ligand-based drug design methods. In the structure-based drug design method, three dimensional structural information of a drug target interacting with small molecules is used to guide drug discovery, whereas the ligand-based method uses information about known ligands of a drug target of interest.

## 12. Molecular Modeling Based Studies

The efforts to discover more efficient and selective HDAC inhibitors have been continually intensified ever since HDAC inhibitors were found active in various clinical trials. Computer modeling has played a critical role in understanding the enzyme-drug interactions, identifying potent inhibitors and obtaining quantitative structure activity relationships of HDAC inhibitors. To this end, both ligand and structure based drug design methods have been employed. For example, in an effort to optimize the structural analogues of cyclopeptide FR235222, an HDAC inhibitor, molecular docking studies were conducted with *cis* and *trans* isomers of 10 analogues of FR235222 and a homologous protein of HDAC1 [180]. This study provided possible bioactive conformation and revealed the contribution of hydrophobic interactions to the stability of the complex. Molecular docking is a rapid process to predict the bioactive conformation of a compound in the active site of a target protein. This method is routinely used to gain insight into the interaction between the enzyme and its inhibitors, especially when the crystal structure of the complex is not available. Towards this goal, several docking studies have been reported in the literature [181,182,183]. The structural details obtained from docking studies can be used for guiding structural modifications of the inhibitors to discover more potent and specific inhibitors for different isoforms of HDAC, or for rational drug design. The same strategy can also be applied to the HDAC inhibitors that are in clinical trials for guiding structural modification to make the drug more potent and isoform specific HDAC inhibitors with potentially reduced toxicities.

For the structural modification, computer-aided scaffold replacement method can be used wherein a portion of the molecule could be replaced, or a group might be added to achieve a particular polar or steric interaction that might enhance the binding affinity. Molecular dynamics (MD) simulation is a computer method to mimic atomic and molecular interactions and observe the structural fluctuations with respect to time. Molecular dynamics simulations of chemically diverse HDAC inhibitors (SAHA, PCI-34051 and C16) and the HDAC isoforms (8, 10 and 11) of the three different classes of zinc-dependent enzyme were also done [184]. The best binding poses from the docking studies were used as the initial structures in the 5 ns MD simulations. MD simulations provided an insight into the interactions between the HDAC and the inhibitor at the molecular level. From this study, it was found that the experimental activities are mainly determined by hydrogen bonds formed by the inhibitor particularly by the metal binding part of the inhibitors and aromatic interactions observed at the tunnel and surface of the active site. Also, the calculated non-bonded interaction energies between the inhibitor and catalytic residues revealed that the subtle difference in the amino acids at the highly conserved active sites of HDAC isoform (M274 in HDAC8, E272 in HDAC10 and L268 in HDAC11) is responsible for the selectivity observed in different HDAC inhibitors. The importance of conserved tunnel forming amino acids and their influence in maintaining the integrity of the tunnel in respective isoforms were also studied by 5 ns MD simulations of wild type HDAC8, 10, and 11, and two mutants (L268M and L268E) of HDAC11 [185]. Another MD simulation study showed continuous opening and closing of hydrophobic active site channel (HASC), affecting the affinity of valproic acid to the HASC [186]. At the same time, the affinity of valproic acid toward the HASC was consistently higher than that obtained for the catalytic site, which suggested that the HASC could be involved in the mechanism of inhibition. Similarly, several MD simulation studies have been conducted to explore the structural and dynamic characterizations of different isoforms of HDAC and specific inhibitors [187,188]. Thus MD simulations of HDAC enzyme in complex with the HDAC inhibitors, especially those made to clinical studies, can aid in understanding the mechanism of action.

Ligand and structure based virtual screening (VS) techniques are also widely used in finding inhibitors of HDACs. Virtual screening is a computer-based method to process compounds from small molecule databases and to identify compounds that are likely to inhibit the biological activity of a particular therapeutic target. Compounds selected by this method should yield higher proportion of active compounds, than a random selection of the same number of molecules. Ligand and structure based VS methods were employed to identify novel non-hydroxamate HDAC inhibitors from the NCI2000 and Maybridge databases [189]. Based on a hit molecule identified by the VS method, three series of compounds were synthesized and evaluated for both HDAC1 inhibitory activity and cytotoxicity to human breast adenocarcinoma MCF-7 cells and human umbilical vein endothelial cells (HUVEC) [189]. Virtual screening against an HDAC6 homology model using the Maybridge database had identified a new HDAC6 selective inhibitor and a carbamate derivative that acts as a prodrug in cell culture, for hydroxamate derived HDAC inhibitors [190]. Similarly, another *in silico* screening of a database containing 167,000 compounds identified one compound with an IC_50_ of 1.6 μM against HDAC8 [191]. By means of virtual screening with docking simulations, six novel HDAC inhibitors with IC_50_ values ranging from 1 to 100 μM were identified [192]. These inhibitors were structurally diverse and had various chelating groups for the active site zinc ion, including *N*-[1,3,4]thiadiazol-2-yl sulfonamide, *N*-thiazol-2-yl sulfonamide, and hydroxamic acid moieties. In fact, a number of studies have used VS as a supporting tool for identifying potential inhibitors of a given enzyme for other diseases as well. For example, a potential inhibitor of *Schistosoma mansoni* HDAC8 (smHDAC8) for the treatment of schistosomiasis, a parasitic disease caused by blood flukes of the genus *Schistosoma*, was identified by screening the Enamine purchasable compound library [193]. The molecules exhibited an inhibitory effect on smHDAC8, and had the capacity to induce apoptosis and mortality in schistosomes.

Synthesis of several putative structures and arriving at a clinically important therapeutic agent involves arduous and careful procedures. At the same time, high-throughput screening of chemical libraries is expensive and resource intensive. Under such conditions, virtual screening of chemical libraries provides an alternative approach to finding active chemical entities and structural scaffolds for the development of novel cancer therapeutic agents. The inexpensive virtual screening method employs either a target based or a ligand based approach. The target based approach uses molecular docking procedure. Since the crystal structures of many isoforms of HDAC are already available, the target based screening can easily be carried out. Homology models of HDAC enzymes can also be used if the crystal structure of a specific isoform is unavailable. For our advantages a number of free small molecule libraries are available for screening, which can later be purchased for testing *in vitro* and *in vivo* studies.

Ligand based virtual screening detects the most effective biologically active lead compound by searching for compounds that have structural or topological similarity or pharmacophoric similarity to a biologically active compound. In our laboratory, using shape-based screening, we have succeeded in finding a lead molecule that led to the discovery of thiazole derivatives as novel inhibitors of metastatic cancer cell migration and invasion [194,195]. Ligand based virtual screening method has proven to be successful in other studies as well [196,197]. Shape based screening is capable of identifying new lead with similar shape as well as electrostatic properties to a lead query molecule. Using HDAC inhibitors that are already in clinical trials as the lead query molecules, shape based screening can identify new structural scaffolds with entirely different zinc binding domain, linker, or cap groups. Indeed, using this approach we have identified several hundreds of potential candidates with various structurally distinct zinc binding domain, linker and cap groups that are currently being tested by HDAC inhibitor assays. 

Docking and energy-optimized pharmacophore mapping of several known HDAC inhibitors identified structural variants that are significant for interactions against Class I HDAC enzymes [198]. Apparently inhibitors with at least one aromatic ring in their linker regions showed higher affinities towards the target enzymes, whereas inhibitors without any aromatic rings were poor binders. In this method the ligand-based pharmacophore modeling and structure based protein-ligand docking are combined to rapidly screen small molecule libraries. The energy descriptors obtained from docking are mapped on to pharmacophore feature sites, which allows the sites to be quantified and ranked on the basis of the energetic terms. In the end this method leads to a final energy minimized pharmacophore hypothesis. Thus this method offers the advantages of both structure-based and ligand-based drug design methods. The same protocol was also used in identifying structural variations that regulate the interaction of HDAC inhibitors against Class II HDAC enzymes [199]. It was shown that inhibitors possessing higher number of aromatic rings in different structural regions might function better. A docking-enabled pharmacophore model also identified HDAC8 inhibitors as anticancer agents [200]. In this study, the best docked conformations of each training set compounds were used for the pharmacophore generation and the best pharmacophore model was then used in database screening to identify novel virtual leads.

## 13. Quantitative Structure Activity Relationship of HDAC Inhibitors

Synthesis of chemical compounds is a costly and resource intensive process. Hence estimation of chemical compounds’ property and/or activity towards a particular enzyme before their synthesis is highly desirable. In this regard, computer-modeling based quantitative structure activity relationship (QSAR) provides a convenient method to predict activity or properties of the molecules of interest. Because of its significant contribution in the drug discovery field, application of both 2D-QSAR and 3D-QSAR modeling has become an integral part of the drug discovery process. For example, 3D QSAR relationships of a series of lactam-based HDAC inhibitor were used for further evaluation of novel lactam-based HDAC inhibitors [201]. This study suggested that HDAC inhibitors which are small in overall size but possess big surface areas with stabilized aromatic cap groups would show better HDAC inhibitory activities [201]. QSAR studies also helped in the design, synthesis and biological evaluation of γ-lactam-based HDAC inhibitors [202,203]. By introducing different cap groups, such as phenyl, naphthyl, and thiophenyl, it was observed that hydrophobic and bulky cap groups can increase the potency of HDAC inhibition because of the hydrophobic interaction between the HDAC and γ-lactam inhibitor. Similarly methoxy and trifluoromethyl substitutions at the *ortho*-, *meta*-, *para*- position of the cap group showed increased HDAC inhibition when the substituent (trifluoromethyl) is more lipophilic. Thus lipophilicity increases the hydrophobic interaction between the surface of HDAC active site and HDAC inhibitor, which in turn improves the HDAC inhibitor activity [203]. A recent survey of published QSAR studies of HDAC inhibitors revealed that the lipophilicity is one of the most important determinants of anticancer activity [204]. Structure activity relationship studies that included the linker region and the surface recognition group to optimize HDAC inhibition identified two lead compounds that are potent inhibitors of HDAC6 and HDAC8, but inactive against HDAC1 [205]. In SAHA-like HDAC inhibitors, a triazole ring that joins the surface recognition cap group to the linker group has shown differential inhibition against HDACs [206]. Structure activity relationships of such triazole-linked hydroxamates displayed a cap group-dependent preference for either five or six methylene spacer groups, and showed several fold greater potency than SAHA. Thus the QSAR studies greatly aid in understanding the factors that affect the biological activity, which can then be applied in rational drug design.

All the molecular modeling techniques described here, including the QSAR studies, provide excellent opportunities to identify potential HDAC inhibitors, either using the known HDAC inhibitors or from scratch, and to guide the structural modifications in the synthesis of novel HDAC inhibitors.

## 14. Concluding Remarks

HDAC inhibitors represent a promising class of anticancer agents, with three of them now approved for cutaneous and/or peripheral T-cell lymphoma. Many HDAC inhibitors are in different stages of clinical trials for various haematological and solid tumors. While HDAC inhibitors alone have displayed anticancer activities in various cancers, a growing number of studies have demonstrated more efficient and tumor specific anticancer activities of HDAC inhibitors when they are given in combination with other drugs. Even though vorinostat, romidepsin and belinostat are approved for cutaneous and/or peripheral T-cell lymphoma, these drugs are still being studied in clinical trials for other types of cancers, either as single agents or in combination with other drugs. This clearly underscore the potential of HDAC inhibitors in cancer treatment. Besides the promising effects on anticancer activities, the use of HDAC inhibitors in other diseases, such as intestinal fibrosis, autoimmune, inflammatory diseases, metabolic disorders and many more, is also growing.

Though there are different structural classes of HDAC inhibitors, the most common HDAC inhibitors are derivatives of four structural classes; hydroxamic acid, benzamide, short chain fatty acid or cyclic peptides. The pharmacophores of these molecules include a metal-binding moiety, a surface binding moiety and a linker connecting them. Presence of an aromatic ring in the linker region seems to enhance the affinity towards the target enzyme. Similarly, hydrophobic and bulky cap groups that bind to the surface region in the HDAC can increase the inhibitor potency. In general, lipophilicity plays an important role in determining the anticancer activity of HDAC inhibitors.

Having discovered the clinical benefits of HDAC inhibitors in various diseases, especially in cancers, there is an increasing need to develop more potent and tumor-specific HDAC inhibitors. However, disruption of multiple pathways by these inhibitors and the lack of specificity of these inhibitors to a target enzyme could contribute to the cytotoxicities that were found in many of the clinical trials. Computational modeling tools, such as docking and molecular dynamics simulations, provide an alternative ways to look into the molecular level interactions between the target enzyme and the inhibitors. Structure activity relationship determines chemical groups in a drug molecule that are responsible for evoking the biological activity of a target enzyme. A combination of docking, molecular dynamics simulations, structure activity relationships and pharamacophore models greatly assist in developing more potent and enzyme specific HDAC inhibitors. Virtual screening has also assisted in finding inhibitors for a specific HDAC enzyme. Both target based and ligand based virtual screening methods are recommended for identifying novel isoform specific HDAC inhibitors. Ligand based virtual screening that identifies new leads with similar shape and electrostatic properties to a lead query molecule, including HDAC inhibitors that are in clinical studies or found in nature, is another way to extract new structural classes of HDAC inhibitors as anticancer agents. Scaffold replacement method is another highly suitable approach by which different pharmacophore regions, such as the zinc binding domain, linker and cap region, in known HDAC inhibitors including those in clinical studies can be modified to synthesize more potent and specific HDAC inhibitors. Computer modeling has emerged as a powerful complement to the experimental approach to finding more potent and specific HDAC inhibitors. As such, clinical studies in combination with basic biological research and computer modeling should enable us to discover a greater variety of HDAC inhibitors specific for a given target, and also to develop tumor specific HDAC inhibitors. This review highlights the interplay between computer modeling based research and experimental research that is essential for the development of novel HDAC inhibitors as anticancer agents.
